# Deletion of the *Capn1* Gene Results in Alterations in Signaling Pathways Related to Alzheimer’s Disease, Protein Quality Control and Synaptic Plasticity in Mouse Brain

**DOI:** 10.3389/fgene.2020.00334

**Published:** 2020-04-09

**Authors:** Wenyue Su, Qian Zhou, Yubin Wang, Athar Chishti, Qingshun Q. Li, Sujay Dayal, Shayan Shiehzadegan, Ariel Cheng, Clare Moore, Xiaoning Bi, Michel Baudry

**Affiliations:** ^1^Graduate College of Biomedical Sciences, Western University of Health Sciences, Pomona, CA, United States; ^2^Key Laboratory of the Ministry of Education for Coastal and Wetland Ecosystem, College of the Environment and Ecology, Xiamen University, Xiamen, China; ^3^Sackler School of Biomedical Sciences, Tufts University, Boston, MA, United States; ^4^College of Osteopathic Medicine of the Pacific, Western University of Health Sciences, Pomona, CA, United States

**Keywords:** calpain-1, brain, signaling pathways, Alzheimer’s disease, long-term potentiation

## Abstract

Calpains represent a family of calcium-dependent proteases participating in a multitude of functions under physiological or pathological conditions. Calpain-1 is one of the most studied members of the family, is ubiquitously distributed in organs and tissues, and has been shown to be involved in synaptic plasticity and neuroprotection in mammalian brain. Calpain-1 deletion results in a number of phenotypic alterations. While some of these alterations can be explained by the acute functions of calpain-1, the present study was directed at studying alterations in gene expression that could also account for these phenotypic modifications. RNA-seq analysis identified 354 differentially expressed genes (DEGs) in brain of calpain-1 knock-out mice, as compared to their wild-type strain. Most DEGs were classified in 10 KEGG pathways, with the highest representations in Protein Processing in Endoplasmic Reticulum, MAP kinase and Alzheimer’s disease pathways. Most DEGs were down-regulated and validation of a number of these genes indicated a corresponding decreased expression of their encoded proteins. The results indicate that calpain-1 is involved in the regulation of a significant number of genes affecting multiple brain functions. They also indicate that mutations in calpain-1 are likely to be involved in a number of brain disorders.

## Introduction

Calpains represent a family of calcium-dependent proteases, which participate in the regulation of a variety of functions under physiological and pathological conditions. Among the 15 members of the family, calpain-1 and calpain-2 are ubiquitously expressed in mammalian cells and have been the most extensively studied (Baudry and Bi, 2016). Significant progress in our understanding of the specificity of calpain-1 functions has been the result of studies conducted with calpain-1 knock-out (KO) mice. These mice are viable and fertile and, at least superficially, appear to be normal. Over the last 7 years, our laboratory has used these mice to analyze the roles of calpain-1 in various brain functions. These studies have provided clear evidence that calpain-1 plays a critical role in synaptic plasticity and learning and memory, as well as in the maturation of the CNS. Thus, calpain-1 KO mice are impaired in theta burst stimulation- (TBS) induced long-term potentiation (LTP) of synaptic transmission in hippocampus and in various forms of learning and memory (Liu et al., 2016; Heysieattalab et al., 2019). They also exhibit a mild form of cerebellar ataxia resulting from the immaturity of the synaptic contacts between the parallel fibers and the Purkinje neurons in cerebellum (Wang et al., 2016a). Similar results were found in the Russel terrier dogs (Forman et al., 2013) and in humans with null mutations in the calpain-1 gene (Gan-Or et al., 2016). Furthermore, these mice are more susceptible to neuronal damage in mouse models of acute glaucoma and traumatic brain injury, indicating that calpain-1 has an additional neuroprotective function (Wang et al., 2016b, 2018).

Calpain-mediated cleavage has been observed in cytoskeleton proteins, membrane-associated proteins, receptors/channels, scaffolding/anchoring proteins, and protein kinases and phosphatases (Wu and Lynch, 2006; Liu et al., 2008; Baudry and Bi, 2013) as well as in transcription factors (Nanduri et al., 2009; Baud and Derudder, 2011). While we have identified a number of calpain-1 targets that could be involved in some of the functions regulated by calpain-1, including PHLPP1, SCOP, and RhoA (Wang et al., 2013; Briz et al., 2015), it is likely that changes in transcription could also be involved in the phenotypic alterations observed in calpain-1 KO mice. A recent study has analyzed changes in muscle transcriptome (Oliver et al., 2018) and identified 55 genes differentially expressed in the quadriceps of calpain-1 KO mice. Analysis of the pathways modified in the knock-out mice provided supportive evidence that the observed phenotypic changes related to skeletal muscles were due to changes in gene expression. We were therefore interested to analyze changes in brain transcriptome in calpain-1 KO mice to determine whether the observed phenotypic changes related to brain function could also be explained, at least partially, by changes in transcriptome. Our results indicate that calpain-1 regulates a much wider set of genes in brain than in muscle, and that these genes belong to signaling pathways involved among others in protein quality control and Alzheimer’s disease.

## Materials and Methods

### Animals

Animal use in all experiments followed NIH guidelines and all protocols were approved by the Institution Animal Care and Use Committee of Western University of Health Sciences. Calpain-1 KO mice on a C57BL/6 background were obtained from a breeding colony established from breeding pairs provided by Dr. Chishti (Tufts University). C57BL/6 mice were purchased from Jackson Labs and were the corresponding WT.

### RNA Isolation, RNA-Seq Library Construction and Sequencing

Whole brains of 2–4-month old male WT and Calpain-1 KO mice were collected, and immediately frozen in liquid nitrogen and stored at −80°C. Total RNA was extracted by TRIZOL (Invitrogen) and DNA was removed by DNaseI (New England Biolabs) according to the manufacturer’s instructions. Quantity and quality of RNA samples were assessed by the NanoDrop spectrophotometer (Thermo Scientific) and Agilent 2100 Bioanalyzer (Agilent Technologies). The six RNA-seq cDNA libraries were constructed by using NEBNext UltraTM II RNA Library Prep Kit according to manufacturer’s protocol (New England Biolabs), and sequenced on Illumina HiSeq 4000 by the pair-end 150-bp sequencing method at Novogene Corporation, Beijing, China).

### RNA-Seq Data Analysis

The raw reads from six RNA-seq datasets used the FASTX-Toolkit^1^ to remove low quality reads and provided the clean reads for further analysis. The clean reads were mapped to *M. musculus* genome (MM10 version of *M. musculus* from UCSC) by HISAT (Pertea et al., 2016). The raw read counts for each gene in each sample were calculated by HTseq (Anders et al., 2015), and we then built a data frame to identify differently expressed gene by DEseq2 between KO and WT, *p* values are adjusted for multiple testing by the Benjamini and Hochberg procedure (Love et al., 2014). Genes with an absolute value of Log_2_FoldChange (KO/WT) > 0.1 and adjusted *p*-value < 0.05 were considered as significantly differentially expressed genes (DEGs). We also considered genes with absolute value of Log_2_FoldChange (KO/WT) > 0.1, *p*-value < 0.0024 and adjusted *p*-value of NA as DEGs due to the independent filtering performed in DEseq2. GO (Gene Ontology), and KEGG (Kyoto Encyclopedia of Genes and Genomes) pathway enrichment analysis for DEGs was performed by KOBAS 3.0^2^ (adjusted *p*-value < 0.05).

### Identification of Transcription Factors (TFs) and TF Target Prediction

Transcription factor genes were identified by mapping the 354 DEGs to TRRUST v2 (Han et al., 2017) mouse gene database, and their target genes were then screened.

### Cell Specific Expression Analysis (CSEA)

In order to identify potential cell specific expression of some of the DEGs, we mapped the DEGs to the currently available dataset provided by the Dougherty lab, using the pSI identification method (Dougherty et al., 2010; Xu et al., 2014; Lakatos et al., 2017)^3^. We applied the CSEA to the 354 DEGs and selected the gene list provided for mice.

### RT-qPCR

We used 1 μg of total RNA as template, after reverse transcription of total RNA performed using the High-Capacity cDNA Reverse Transcription Kit (Thermo Fisher Scientific) according to the manufacturer protocol; RT-qPCR was conducted with Fast SYBR Green Master Mix (Thermo Fisher Scientific) by following the manufacturer’s instruction on a CFX 96 Real-time RCR platform (Bio-Rad). Data were acquired in independent biological triplicates. Relative gene expression was calculated based on 2^–ΔΔ*Ct*^ method using *Gapdh* as an internal control. All primer sequences of selected genes are listed in Supplementary Table S1.

### Brain Homogenate Preparation and Western Blot Analysis

Whole brains were homogenized in RIPA buffer with protease inhibitors at 4°C. After centrifugation at 13,000 × *g* at 4°C for 15 min, protein amounts in the supernatant were quantified using the BCA Assay kit (Pierce Biotechnology). Proteins from whole brains of WT and calpain-1 KO mice were subjected to 10% SDS-PAGE, and proteins were transferred to a PVDF membrane with 100 V for 1 h at 4°C. After blocking for 2 h at room temperature with 3% bovine serum albumin in TBS buffer, membranes were incubated at 4°C overnight with rabbit anti-HSPA1B (1:500; PA5-28369; Thermo Fisher Scientific), anti-DNAJB1 (1:1000; 13174-1-AP; Proteintech), anti-Insulin degrading enzyme/IDE (1:1000; ab32216; abcam), anti-PLA2G4E (1:200; 18088-1-AP; Proteintech), anti-NGFI-B alpha/Nur77/NR4A1 (1:1000; NB100-56745; Novus Biologicals), anti-PER2 (1:300; ab180655; Abcam) antibodies and mouse anti-ARC (1:500; sc-17839; Santa Cruz) antibody. After incubation in primary antibodies, membranes were washed with TBST buffer and incubated for 2 h at room temperature with IRDye 680RD goat anti-rabbit (1:10,000; LI-COR Biosciences) and IRDye 800CW goat anti-mouse (1:10,000; LI-COR Biosciences). Thereafter, membranes were washed 3 times with the TBST and 1 time with TBS buffer. Immunoreactivity was detected with the LI-COR Odyssey system (LI-COR Biosciences).

### Immunohistochemistry

Frozen sections of hippocampal slices were prepared as described previously (Wang et al., 2014). Sagittal sections (20-μm thick) of the brain were cut on a cryostat and processed for blocking for 1 h at room temperature with 10% goat serum in PBST buffer; immunohistochemistry was performed with overnight incubation at 4°C with anti-HSPA1B (1:100), anti-DNAJB1 (1:100), anti-Insulin degrading enzyme/IDE (1:200), anti-PLA2G4E (1:100), anti-NGFI-B alpha/Nur77/NR4A1 (1:200), anti-PER2 (1:200), anti-ARC (1:50; sc-15325; Santa Cruz) and anti-Doublecortin (1:100; sc-8066; Santa Cruz) antibodies. Sections were then washed 3–5 times with PBS and incubated with Alexa Fluor 594 goat anti-rabbit IgG, Alexa Fluor 594 goat anti-mouse IgG, Alexa Fluor 594 donkey anti-goat IgG and/or Alexa Fluor 488 goat anti-rabbit IgG (1:400, Invitrogen) secondary antibodies for 2 h at room temperature. Fluorescence images were captured with a Zeiss laser scanning confocal microscope (Zeiss) and analysis of fluorescent signals was carried out by using ZEN (Zeiss) software.

### Co-expression Network

In order to understand the interactions between calpain-1 and the DEGs identified in this study, we used the GeneMANIA database to perform a co-expression network analysis (Warde-Farley et al., 2010). After selection of *Mus musculus* as the organism, genes coding for calpain-1 and selected proteins were entered into the search bar.

### Statistical Analysis

All data are presented as means ± SD. Unpaired *t*-test in GraphPad Prism (version 7.0) was carried out to analyze significance; the difference between WT and calpain-1 KO mice was considered to be significant at *p* < 0.05.

## Results

### Transcriptomic Analysis of Brains From WT and Calpain-1 KO Mice

**FIGURE 1:**
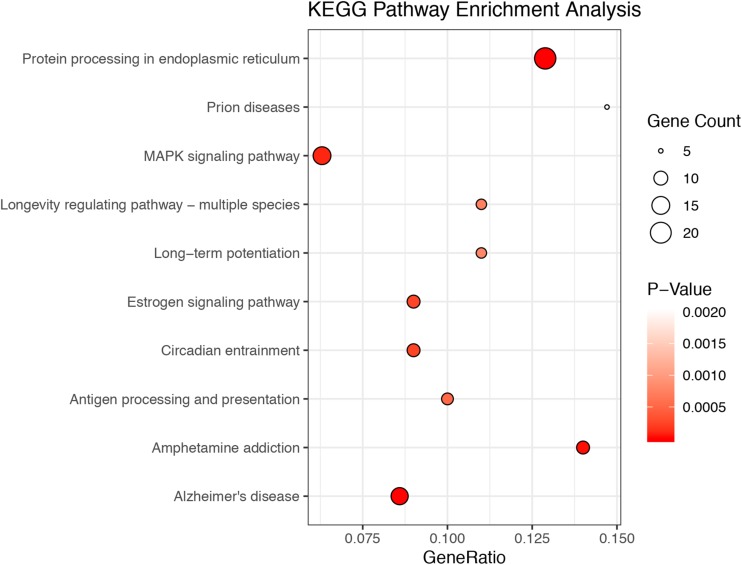
The top 10 enriched KEGG pathways among DEGs. The 354 DEGs belong to 39 KEGG pathways. The figures depict the number of DEGs in the top 10 most enriched pathways, with the size of the circles related to the number of genes in each pathway.

**FIGURE 2:**
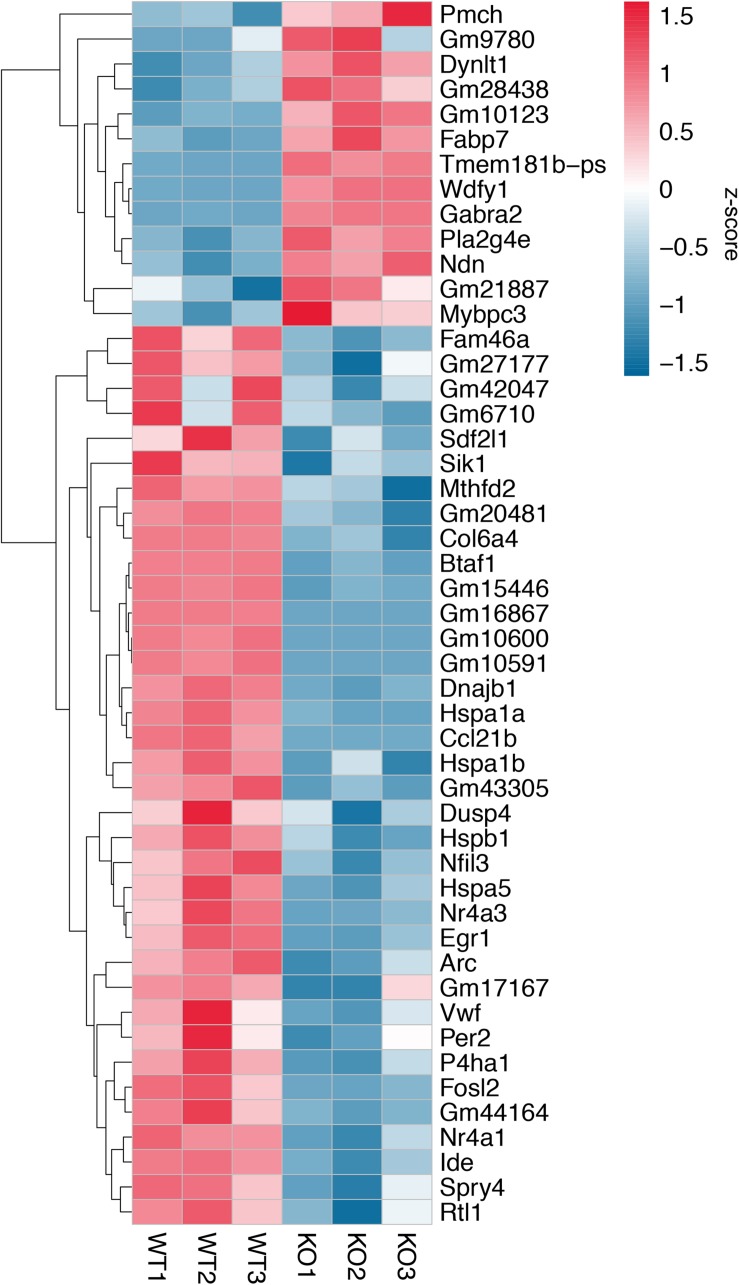
Gene expression patterns for 49 DEGs. Changes in gene expression for 49 DEGs with adjusted *p*-value **<** 0.05, *p*-value < 0.0024, and |log2FoldChange (KO/WT)| > 0.5. Each column represents a separate animal, and changes in gene expression are color-coded with the scale bar on the right of the figure.

A total of 20.87–27.37 millions of 150 bp-end reads were generated from all the samples using RNA sequencing (Table 1). After filtering low quality reads, high-quality reads were aligned to *Mus musculus* mm10 genome, where the average percentage of read mapping in WT and KO was 94.76 and 94.88%, respectively (Table 1). We used DEseq2 to normalize gene expression and performed clustering analysis for all expressed genes in the samples. Consistent expression patterns were found among the three replicates (KO or WT), suggesting good repeatability (Supplementary Figure S1 and Supplementary Table S2). We identified 354 DEGs between WT and calpain-1 KO mice (310 genes with adjusted *p*-value < 0.05 and 44 genes with *p*-values < 0.0024 but filtered out in DEseq2; Supplementary Table S3). The functional enrichment of DEGs identified 346 enriched GO terms (Supplementary Table S4) and 37 enriched pathways (Supplementary Table S5). We used the KEGG pathway analysis to map these DEGs (Supplementary Table S6 and Figure 1). The most represented pathways were the protein processing in endoplasmic reticulum, the MAPK signaling and the Alzheimer’s disease pathways. Other interesting clusters consisted in the amphetamine addiction, the circadian entrainment, the estrogen signaling and LTP pathways. Figure 2 depicts the expression pattern for 49 DEGs with significant changes in calpain-1 KO mice, as compared to WT mice. Note that the majority of these genes are down-regulated in calpain-1 KO mice, with only a small number up-regulated.

**TABLE 1:** Data quality summary.

Sample	Raw Reads	Clean Reads	Q20(%)	Q30(%)	GC Content (%)	Alignment rate to mm10 genome (%)
WT-1	23245619	22829317	96.93	92.56	49.45	94.95
WT-2	27374309	26077875	95.09	89.16	49.58	94.53
WT-3	24181788	23762946	97.00	92.75	49.23	94.79
KO-1	20872134	20492714	96.96	92.67	49.24	94.95
KO-2	25908758	25264932	96.61	92.09	49.24	94.24
KO-3	26448006	25792530	96.48	91.62	49.22	95.44

***Raw reads:** total amount of reads of raw data, each four lines taken as one unit. For paired-end sequencing, it equals the amount of read1 and read2, otherwise it equals the amount of read1 for single-end sequencing. **Clean reads:** total amount of reads from clean data, with each four lines taken as one unit. For paired-end sequencing, it represents the amount of read1 and read2; otherwise, it represents the amount of read1 for single-end sequencing. **Quality cut-off**: 20; Minimum percentage: 60. **Q20, Q30:** (Base count of Phred value > 20 or 30)/(Total base count). **GC content:** (G & C base count)/(Total base count).*

Since our analysis was performed on whole brain, it did not provide information regarding possible differences in DEGs between different brain regions and/or cell types. We performed a cell specific expression analysis (CSEA) by taking advantage of the dataset, which identified genes enriched in different brain regions and cell types. Mapping our DEGs with this dataset could indicate whether some of these genes are indeed more represented in particular brain regions and/or cell types (Supplementary Figure S2). Using this CSEA, a number of DEGs between WT and calpain-1 KO brains were expressed in D1- and D2-positive spiny neurons, cortical immune cells and neurons (Supplementary Figure S2). Interestingly, one of the DEGs is *Nr4a1*, a nuclear receptor involved in the regulation of the dopaminergic system (see below). TFs are major factors in transcriptional regulation by regulating the transcription of their target genes. Among the 354 DEGs we identified 25 TFs, including *Nr4a1*, and some of their target genes were also found in the DEGs (Supplementary Table S7).

### Validation of RNA-Seq Data

**FIGURE 3:**
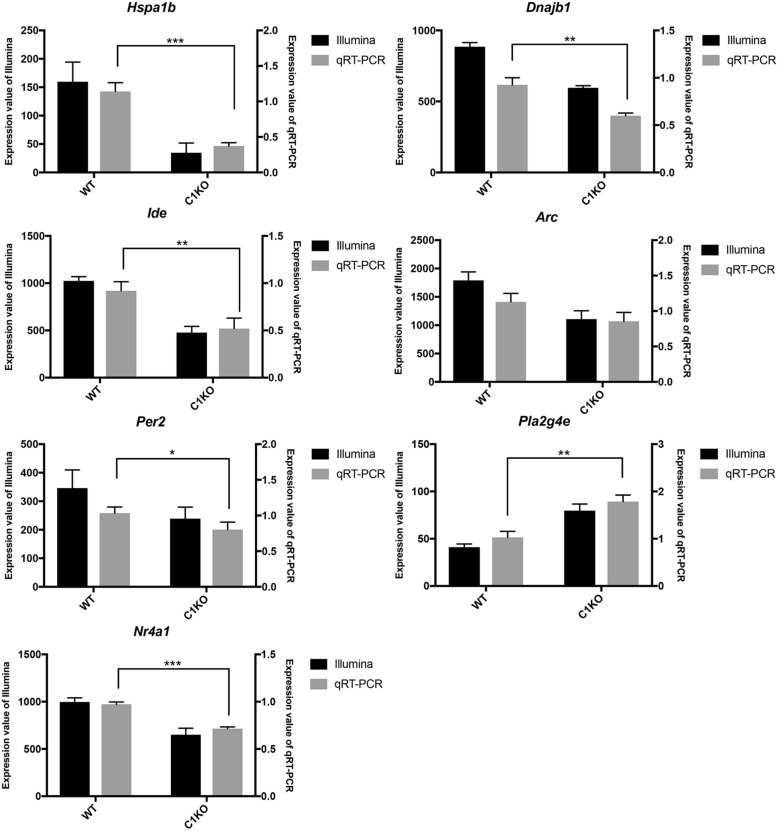
RT-qPCR validation of RNA-seq results in WT and calpain-1 KO mice. RT-qPCR to validate 7 genes (*Hspa1b*, *Dnajb1*, *Ide*, *Arc*, *Per2*, *Pla2g4e*, and *Nr4a1*), which were selected from DEGs by RNA-seq. The black bars represent the expression levels determined by RNA-seq (left *y*-axis) and gray bars represent relative expression calculated by RT-qPCR data (right *y*-axis) with the error bars representing standard deviations of three independent biological replicates. Unpaired *t*-test in Prism 7 was used to calculate *p*-values, * *p* < 0.05, **p < 0.01, ***p < 0.001.

To validate the RNA-seq data, we selected several DEGs (Table 2). Heat Shock Protein Family A (Hsp70) Member 1B (*Hspa1b*) and DnaJ Heat Shock Protein Family (Hsp 40) Member B1 (*Dnajb1*) genes are involved in Protein processing in endoplasmic reticulum pathway, Insulin Degrading Enzyme (*Ide*), Activity Regulated Cytoskeleton Associated Protein (*Arc*) and Period Circadian Regulator 2 (*Per2*) genes are related to Alzheimer’s disease, Amphetamine addiction and Circadian entrainment pathways respectively. Phospholipase A2 Group IVE (*Pla2g4e*) and Nuclear Receptor Subfamily 4 Group A Member 1 (*Nr4a1*) genes are involved in MAPK signaling pathway. These genes were selected because of high |log2FoldChange| in their levels analyzed by RNA-seq. Most of these genes were significantly down-regulated in calpain-1 KO mice, except for *Pla2g4e*. We first used RT-qPCR to validate the changes in expression of these 7 genes. As shown in Figure 3, the changes in relative gene expression observed with RT-qPCR were similar to those obtained with the sequencing data, indicating the reliability of RNA-seq data.

**TABLE 2:** List of genes selected for validation at the protein levels.

DEG	Protein Name	KEGG Pathway	Percent Change
*Hspa1b*	HSP70 Member 1B	Protein Processing in ER	−79%
*Dnajb1*	HSP40 Member B1	Protein Processing in ER	−33%
*Ide*	Insulin Degrading Enzyme	Alzheimer’s disease	−54%
*Arc*	Activity-Regulated Cytoskeleton Associated Protein	Amphetamine Addiction	−38%
*Per2*	Period Circadian Regulator 2	Circadian Entrainment Pathway	−31%
*Pla2g4e*	Phospholipase A2 Group IVE	MAPK signaling pathway	+94%
*Nr4a1*	Nuclear Receptor Subfamily 4 Group A Member 1	MAPK signaling pathway	−35%

*These genes were selected for detailed analysis due to the relatively high level of changes between the WT and the calpain-1 KO mice.*

### Changes in Protein Expression in Brains From WT and Calpain-1 KO Mice

**FIGURE 4:**
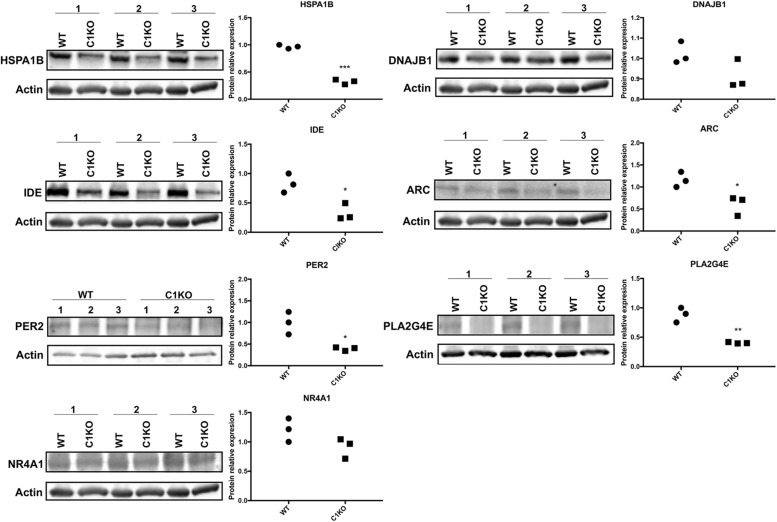
RNA-seq results were validated by Western blot analysis in WT and calpain-1 KO mice. Western blot images and quantifications showing the expression levels of HSPA1B, DNAJB1, IDE, ARC, PER2, PLA2G4E, and NR4A1 proteins in three independent replicates, respectively. Dot plots were used for the quantification of the expression levels of 7 proteins compared to actin control in WT and calpain-1 KO mice. Unpaired t-test in Prism 7 was used to calculate p values, *p < 0.05, **p < 0.01, ***p < 0.001.

We analyzed the levels of the proteins encoded by the genes listed in Table 2 by Western blot analysis (Figure 4). The results of Western blot showed that the expression of HSPA1B, IDE, ARC and PER2 was significantly decreased (*p* < 0.05), while DNAJB1 expression showed no significant change between WT and calpain-1 KO mice. In addition, levels of PLA2G4E decreased in calpain-1 KO, which was the opposite from the RNA-seq and RT-qPCR results.

**FIGURE 5:**
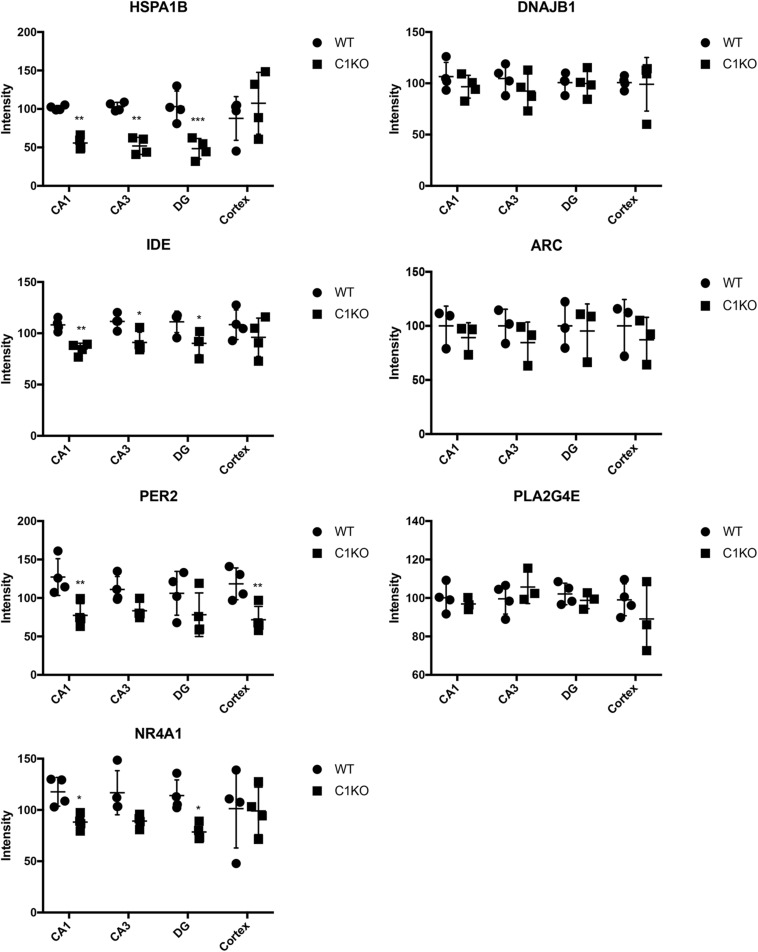
Quantification of immunostaining for 7 proteins in the brain of WT and calpain-1 KO mice. Immunostaining for WT and calpain-1 KO mice was performed in brain slices. The mean fluorescence intensity was quantified in CA1, CA3, DG and cortex. Scatter dot plots were used to represent the expression of 7 proteins in four regions, with the lines in the dot plots representing standard deviations. Unpaired t-test in Prism 7 was used to calculate *p* values, *p < 0.05, **p < 0.01, ***p < 0.001.

Next, we analyzed the expression of those 7 proteins in hippocampus and cortex of WT and calpain-1 KO mice by immunostaining. As shown in Figure 5, immunoreactivity for HSPA1B and IDE in CA1, CA3 and DG from calpain-1 KO mice was significantly lower, as compared with WT, although no change was observed in cortex. The level of PER2 was decreased significantly (*p* < 0.05) in CA1 and cortex, and the level of NR4A1 was decreased significantly (*p* < 0.05) in CA1 and DG regions. However, the results of immunostaining showed that the levels of DNAJB1, PLA2G4E and ARC were not significantly different between WT and calpain-1 KO mice in any brain region analyzed. Representative images are shown in Supplementary Figures S3–S5.

**FIGURE 6:**
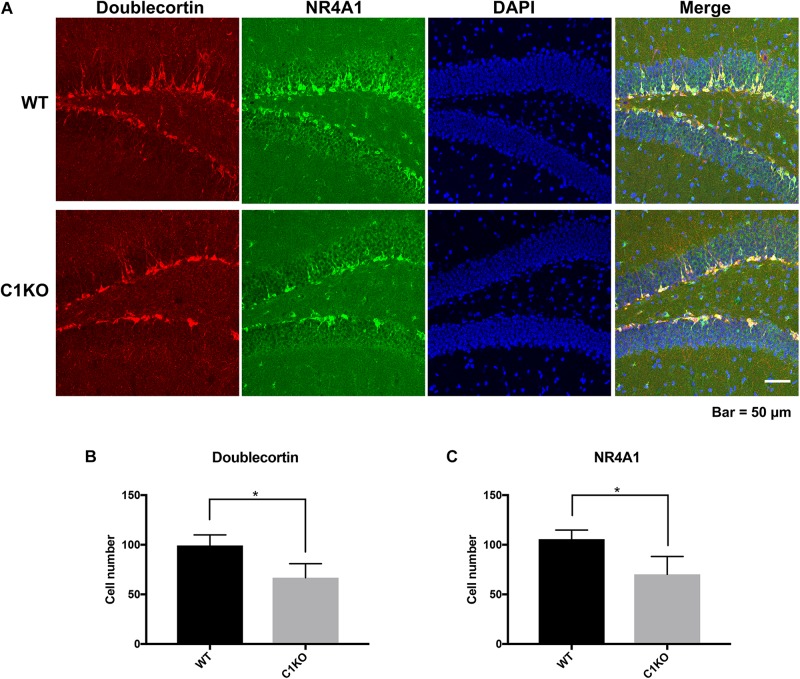
Calpain-1 knockout reduced newly born neurons in the DG of hippocampus. **(A)** Doublecortin and NR4A1 immunofluorescence-double staining in the DG. Red represents Doublecortin. Green represents NR4A1. Blue represents DAPI. **(B)** Quantification of the numbers of cells labeled with doublecortin. **(C)** Quantification of the numbers of cells labeled with NR4A1. The black bars represent WT samples and gray bars represent calpain-1 KO samples with error bars representing standard deviations. *p < 0.05 (Student’s *t*-test).

Because the staining pattern of NR4A1 in the dentate gyrus was reminiscent of that of newly born neurons, we performed double-staining for NR4A1 and doublecortin, a marker of immature progenitor neurons (Figure 6). Interestingly, NR4A1 and doublecortin were highly co-localized in the dentate gyrus, indicating that NR4A1 is indeed localized in immature progenitor neurons. In addition, the numbers of cells labeled with NR4A1 and doublecortin were significantly decreased in calpain-1 KO mice, as compared to WT mice.

## Discussion

Previous studies have reported several phenotypic differences related to various brain functions in calpain-1 KO mice. Thus, we found that these mice exhibit impairment in several forms of synaptic plasticity in the hippocampus and cerebellum and in various forms of learning and memory (Wang et al., 2014; Zhu et al., 2017; Heysieattalab et al., 2019). In addition, we also reported that these mice exhibit cerebellar ataxia (Wang et al., 2016a) and are more susceptible to injury in various forms of acute neuronal insults, including traumatic brain injury (Wang et al., 2016b, 2018). While these phenotypic differences could be explained by the cellular functions of calpain-1 in the regulation of various pathways, we were interested in exploring potential changes in gene expression that could also account for such differences. A recent study identified 55 genes differentially expressed in muscles from the same mice (Oliver et al., 2018), and we analyzed changes in gene expression in brain between calpain-1 KO mice and WT mice. In contrast to the muscle, we found 354 DEGs in brain of calpain-1 KO mice as compared to WT mice. The functional enrichment analysis indicated that these 354 DEGs were significantly enriched in several signaling pathways (Figure 1 and Supplementary Table S5), which play important roles in normal brain functions and are linked to a number of neurological/neuropsychiatric disorders. While the majority of DEGs were down-regulated (270), a significant number (84) were upregulated (Supplementary Tables S4, S5). Among these several belong to the ribosome pathway, which is interesting since alterations of these genes might be involved in Alzheimer’s disease (Li et al., 2018). CSEA also indicated that some of the DEGs were enriched in specific brain regions and/or cell types. We first validated the results of the RNA-seq analysis with RT-qPCR for 7 of the genes and found a very good correlation between the results of these 2 analyses. We further analyzed changes in expression of the proteins encoded by these genes and for the most part found a good correlation between changes in gene expression and changes in protein expression. A notable exception was for *Pla2g4e*, which encodes a member of the PLA2 family. While the expression of the gene was almost doubled, protein expression assessed with Western blot was decreased by more than 50%. In contrast, analysis of this protein by immunohistochemistry in several brain regions did not reveal any difference. Reasons for these discrepancies are not clear, and these results suggest that complex interactions between different calpain-1 targets might be involved in widespread alterations in signaling pathways.

Among the enriched KEGG pathways revealed by the analysis, the protein processing pathway in the endoplasmic reticulum contains many heat-shock protein-associated proteins (Supplementary Table S6), which are important for correct protein processing and trafficking. While calpain has been proposed to participate in various disorders related to abnormal protein folding, including Alzheimer’s disease (Nixon, 2003), Parkinson’s disease (Samantaray et al., 2008), and Huntington’s disease (Lee and Kim, 2006), the specific roles of calpain-1 in these disorders have rarely been addressed. The large decrease in the expression of genes and proteins in this pathway fits well with the neuroprotective function of calpain-1 in the brain and the recent findings that humans with calpain-1 deletion exhibit a variety of forms of ataxia/spasticity (Wang et al., 2016a). Decrease in gene and protein expression of the MAP Kinase pathway would also be expected to have widespread consequences considering that this pathway plays critical roles in many brain processes, including synaptic plasticity and cognition (Adams and Sweatt, 2002) as well as stroke (Sun and Nan, 2016) and glioblastoma (Hannen et al., 2017). Thus, changes in the MAP kinase pathway could also account for the impairment in synaptic plasticity and learning and memory we observed in calpain-1 KO mice. This pathway has also been proposed to participate in anxiety, depression and drug addiction (Chen and Sommer, 2009), and it will be of interest to further explore potential phenotypic differences related to these behaviors in calpain-1 KO mice.

A third pathway affected by calpain-1 deletion is the Alzheimer’s disease pathway, with significant decrease in IDE and PER2. IDE has been discussed as a potential target for both diabetes and Alzheimer’s disease (Pivovarova et al., 2016). The decrease in IDE observed with calpain-1 deletion would suggest that individuals with calpain-1 deletion would show decreased risk for developing AD. On the other hand, since calpain has been implicated in the etiology of AD, our result would suggest that calpain-2 rather than calpain-1 might be implicated in AD. This fits well with a recent report indicating that calpain-2 is overactivated in pre-symptomatic AD (Ahmad et al., 2018). Alterations in the circadian rhythm, including sleep disorders, have been implicated in the development of neurodegenerative disorders, including AD (Hood and Amir, 2017; Wall et al., 2018). Circadian alterations and decreased expression of PER2 have been reported in brain of transgenic model of AD (Wu et al., 2018), and it would be interesting to examine sleep behavior in calpain-1 KO mice. Expression of ARC was relatively low in both WT and calpain-1 KO mice, and while we detected a significant difference in the overall brain levels of ARC, there was no significant differences in ARC levels in hippocampus or cortex. Reasons for this discrepancy are not clear, but *Arc* is an immediate early gene, and expression level could vary rapidly depending on the experimental conditions.

NR4A1 is a transcription factor of the nuclear receptor family and has been found to play numerous functions in the brain, including apoptosis, dopamine neuron survival and regulation of spine density in excitatory neurons (Rajpal et al., 2003; Chen et al., 2014; Rouillard et al., 2018). Down-regulation of NR4A1 would thus be expected to have widespread consequences. Interestingly, we found that NR4A1 was localized in immature progenitor neurons in the dentate gyrus, and that the numbers of such neurons were decreased in calpain-1 KO mice. This result also fits well with the neuroprotective function of calpain-1, as it is possible that there newly born neurons might not be able to survive for extended periods of time in the KO mice. Since neurogenesis has been implicated in learning and memory (Toda et al., 2019), decreases in NR4A1 may also contribute to learning impairment in calpain-1 KO mice. Again, further studies are needed to explore additional phenotypic differences in calpain-1 KO mice related to decreased expression of this gene.

What is the connection between calpain-1 and the regulation of these 354 DGEs? Calpain has been shown to cleave several TFs (Vosler et al., 2008), and it is conceivable that some of the observed differences in gene expression are the results of changes in these TFs. It is also conceivable that lack of calpain-1 mediated truncation alters their functions. Moreover, we identified a number of TFs among the DEGs as well as some of the target genes of these TFs. Additionally, changes in some of these DGEs may also be due to compensational responses. A recent review discusses the roles of calpain during development and although it indicates that calpain plays a role in various aspects of development, including cell division and cell migration, our understanding on its overall function remains very limited (Araujo et al., 2018).

**FIGURE 7:**
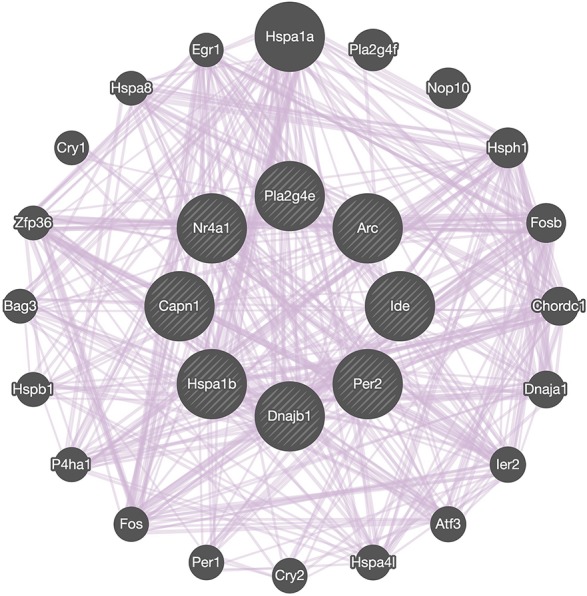
Network analysis. GeneMANIA diagram showing co-expressed interactions among *Capn1* and 7 identified DEGs and their neighbors. The purple lines connecting the genes indicate co-expression. The genes in central circle are *Capn1* and 7 DEGs found in present study. The remaining genes in outer circle are related co-expressed interacting partners.

Co-expression network analysis showed the interactions between *Capn1* and the 7 identified DEGs we selected to analyze in detail (Figure 7). The analysis indicated that all these genes form a network of interacting genes and also revealed another layer of interacting genes. However, co-expression network can only be used for identifying correlations and which genes are active in the same biological processes (van Dam et al., 2017), suggesting that further analysis is needed to better understand the role of calpain-1 in gene regulation.

A surprising finding of our study is that there is a complete lack of overlap in the DEGs in brain and muscle from the same calpain-1 KO mice. We identified 354 DGEs in the brain while Oliver et al. (2018) identified 55 DGEs and they are completely different. Importantly, muscle cells and brain cells are derived from different cell lineages with muscle cells originating in the mesoderm and brain cells in the ectoderm. It is therefore conceivable that an early event during differentiation could account for the differential effects of calpain-1 deletion on gene expression in different cell types.

## Conclusion

Our results underscore the important role of calpain-1 in brain development, synaptic plasticity, protein quality control and in the regulation of neuronal death and in Alzheimer’s disease. They confirm our hypothesis that calpain-1 activation is critical for neuronal survival and several forms of synaptic plasticity and learning and memory. They also provide new avenues of research to further explore the functions of calpain-1 in various disorders, including Parkinson’s and Alzheimer’s disease.

## Data Availability Statement

The raw datasets were deposited into the NCBI database under Sequence Read Archive (SRA) accession number SRP225119.

## Ethics Statement

The animal study was reviewed and approved by IACUC.

## Author Contributions

WS and QZ generated many of the data and contributed to the writing of the manuscript. YW provided tissue samples and RNA preparation and contributed to the writing of the manuscript. AtC provided the mice and was instrumental in getting the study done. QL provided expertise in RNA extraction, RNA-seq and data analysis. SD, SS, ArC, and CM generated some of the data. XB provided expertise in data analysis, data interpretation and contributed to the writing of the manuscript. MB supervised the study, worked on data interpretation and contributed to the writing of the manuscript.

## Conflict of Interest

The authors declare that the research was conducted in the absence of any commercial or financial relationships that could be construed as a potential conflict of interest.

## Footnotes

1 http://hannonlab.cshl.edu/fastx_toolkit/

2 http://kobas.cbi.pku.edu.cn/kobas3/?t=1

3 http://genetics.wustl.edu/jdlab/csea-tool-2/
