# Supplementary material for: Deletion of the Capn1 Gene Results in Alterations in Signaling Pathways Related to Alzheimer’s Disease, Protein Quality Control and Synaptic Plasticity in Mouse Brain
Source: Front Genet. 2020 Apr 9;11:334. doi: 10.3389/fgene.2020.00334 (PMC7161415; doi:10.3389/fgene.2020.00334)
Supplement: Supplementary file 1 [file Presentation_1.pdf]

## Supplemental Material

## Figures S1-S5

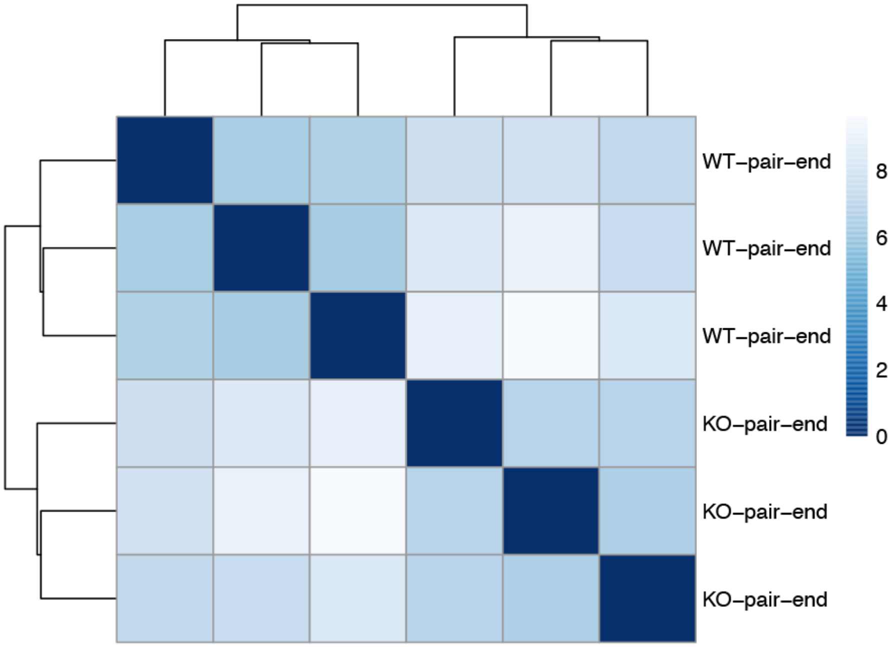


## Figure S1

Clustering analysis showed consistent expression patterns in the three biological replicates of each sample (KO or WT), suggesting good repeatability.

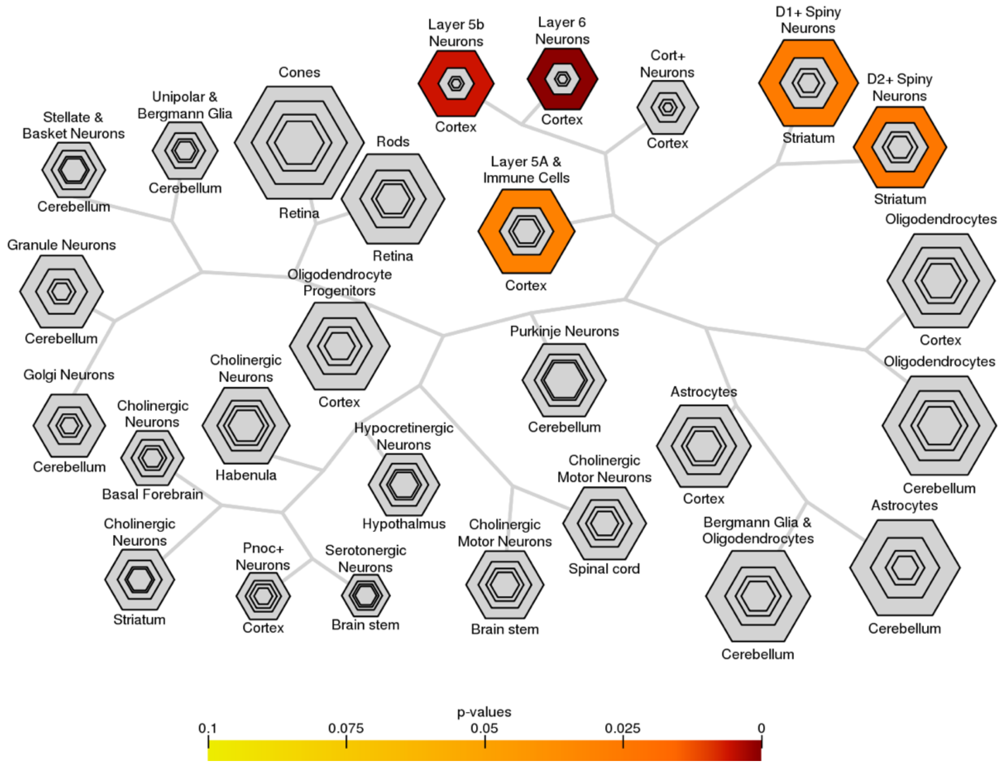


**Figure S2** Cell-type specific expression analysis (CSEA) of 354 DEGs.

CSEA shows that some DEGs are expressed in spiny GABAergic neurons, cortical immune cells and neurons. Different cell types are represented by a multilayer hexagonal and color coded by *p* values (Fisher's exact test) as shown in the bottom scale bar. The hexagon size is scaled to the specific and enriched transcripts number at four stringency thresholds (pSI: 0.05, 0.01, 0.001, 0.0001) and the hexagon with the more stringent threshold (pSI < 0.0001) is in the center (Xu et al., 2014; Lakatos et al., 2017). For example, cortical neurons have smaller *p* value than spiny neurons at pSI < 0.05 level.

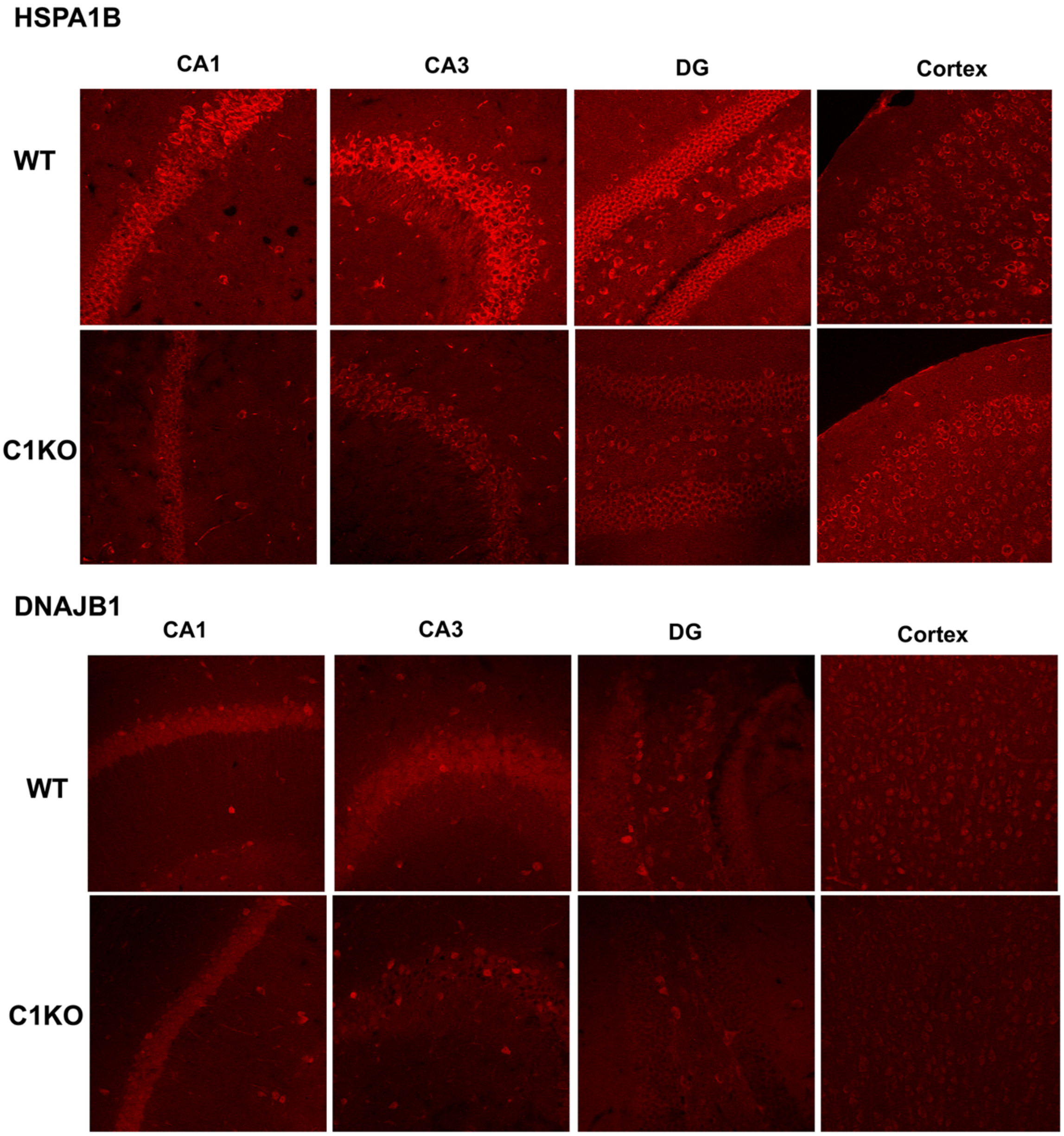


**Figure S3**

Representative images for HSP70 (HSPA1B) and HSP40 (DNAJB1) in hippocampus and cortex from WT and calpain-1 KO mice (C1KO).

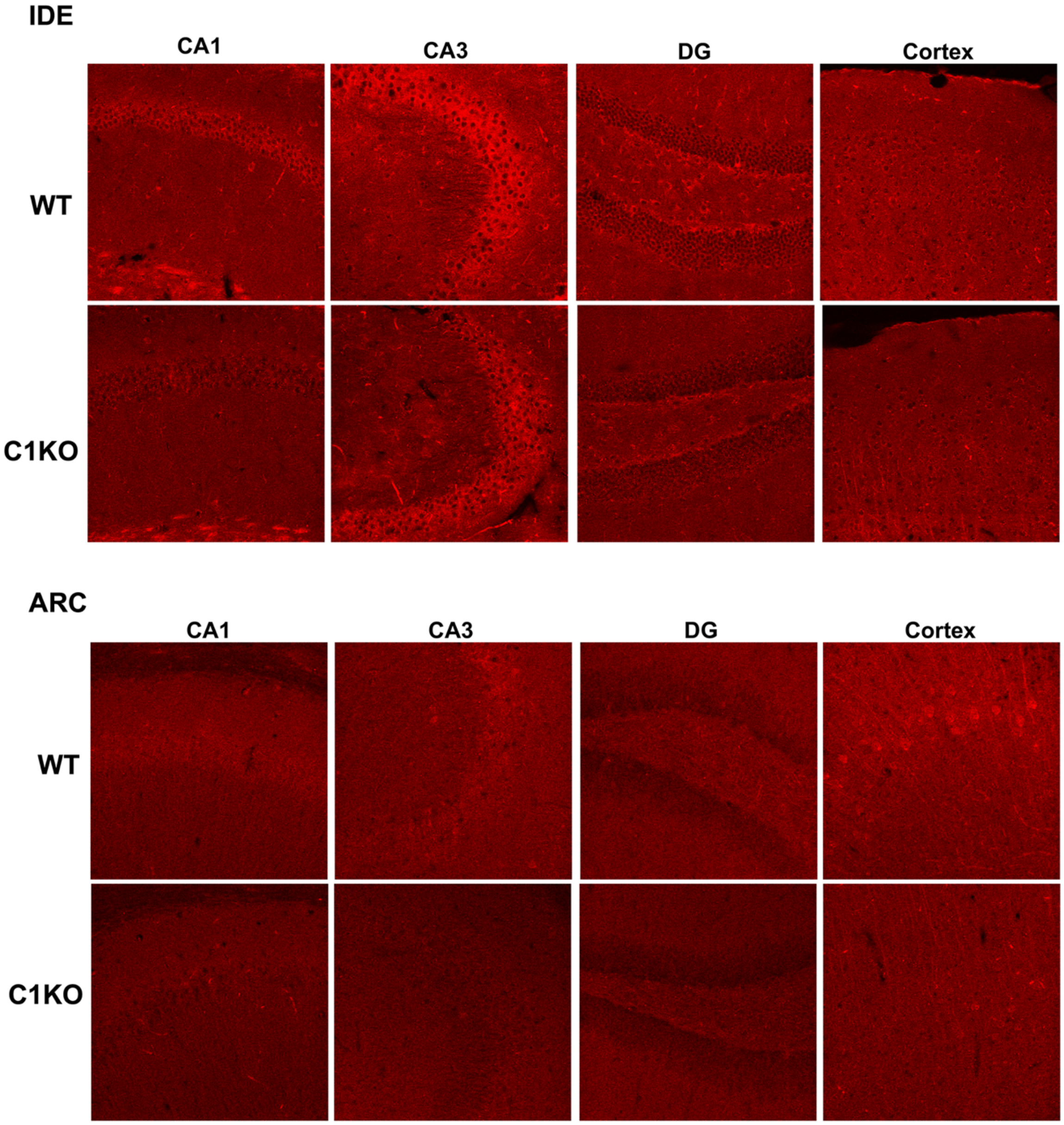


**Figure S4**

Representative images for Insulin Degrading Enzyme (IDE) and Activity-Regulated Cytoskeleton Associated Protein (ARC) in hippocampus and cortex from WT and calpain-1 KO mice (C1KO).

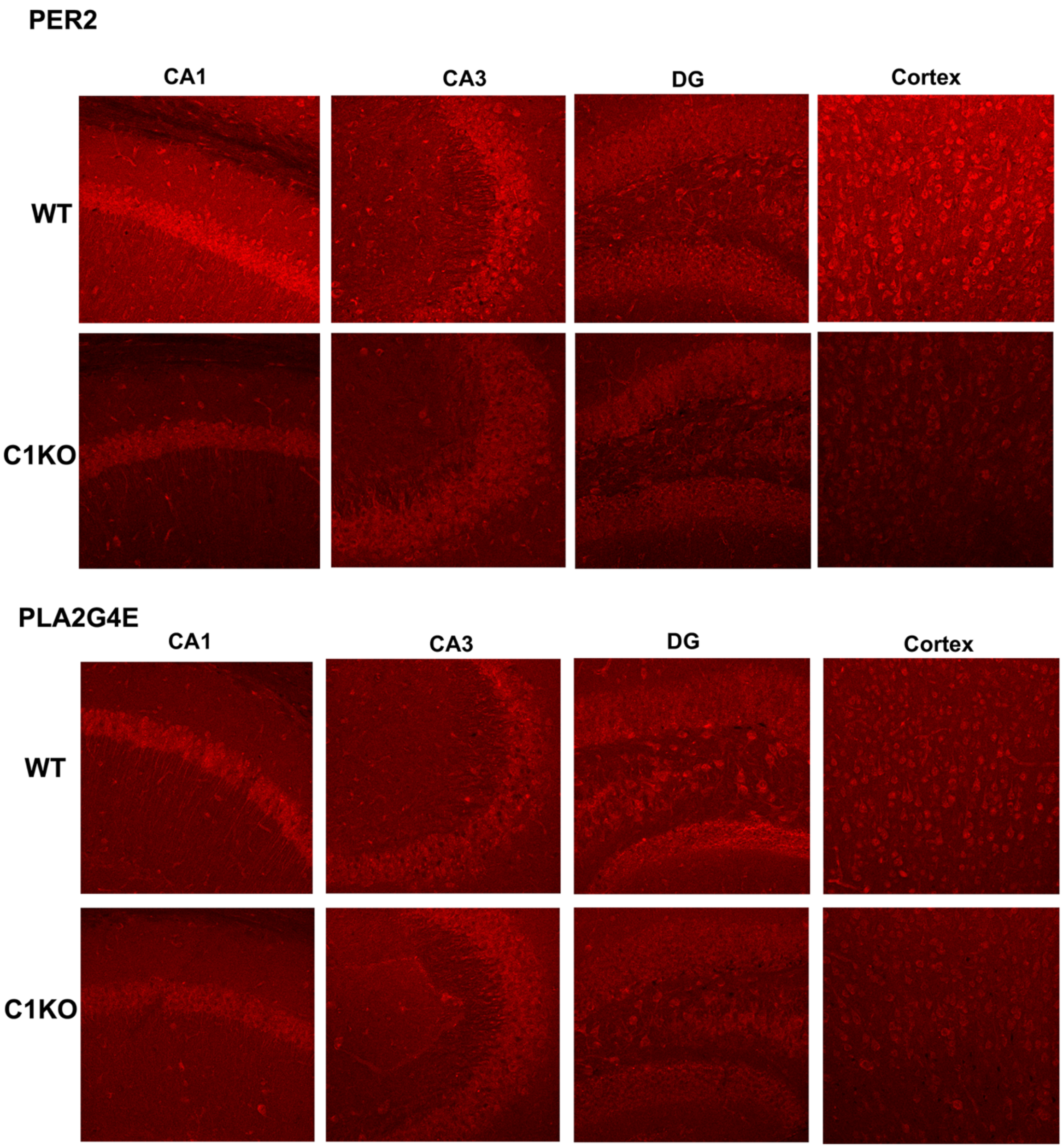


**Figure S5**

Representative images for Period Circadian Regulator 2 (PER2) and Phospholipase A2 Group IVE (PLA2G4E) in hippocampus and cortex from WT and calpain-1 KO mice (C1KO).
